# Differential risk and protective factors for conventional adenomas and serrated lesions: implications for colorectal cancer prevention

**DOI:** 10.3389/fpubh.2026.1949376

**Published:** 2026-10-14

**Authors:** Junjie Cai, Yiliu Huang, Yi Luo, Hui Zeng, Jun Yang, Yingshuang Zhu

**Affiliations:** 1 Department of Epidemiology and Biostatistics, School of Public Health and Nursing, Hangzhou Normal University, Hangzhou, China; 2 Department of Nutrition and Toxicology, School of Public Health and Nursing, Hangzhou Normal University, Hangzhou, China

**Keywords:** colorectal cancer prevention, conventional adenoma, protective factor, risk factor, serrated lesion

## Abstract

Conventional adenomas and serrated lesions are two major precursor lesions of colorectal cancer. They arise through distinct carcinogenic pathways, namely the conventional adenoma–carcinoma sequence and the serrated neoplasia pathway. These pathways differ substantially in their underlying molecular mechanisms, implying that the risk factor profiles for the two types of lesions may be different. However, existing evidence remains fragmented, and an integrated comparative synthesis of these differential associations is lacking. In this review, we synthesize and compare current evidence on the mechanistic characteristics and lifestyle, metabolic, and nutritional determinants of conventional adenomas and serrated lesions. By synthesizing the available evidence on the two precursor pathways, this review may contribute to a better understanding of conventional adenomas and serrated lesions, and offer insights for future research into their distinct risk factors, underlying mechanisms, and implications for colorectal cancer prevention.

## 1 Introduction

Colorectal cancer (CRC) is one of the most common malignancies worldwide. According to the 2022 Global Cancer Statistics, it ranks third in incidence and second in mortality (1). Accumulating evidence has shown that early detection and removal of colorectal precursor lesions through screening can effectively reduce both the incidence and mortality of CRC (2, 3). Conventional adenomas and serrated lesions are the two main precursor lesions involved in colorectal carcinogenesis. Currently, CRC screening strategies mainly focus on the detection and management of conventional adenomas, whereas serrated lesions have received comparatively less attention. However, growing evidence suggests that serrated lesions contribute substantially to interval CRC and may account for approximately 15–30% of CRC cases (4).

The different levels of attention given to conventional adenomas and serrated lesions are closely related to their distinct pathological characteristics and historical understanding. Conventional adenomas, estimated to account for 60–80% of CRC precursor lesions, are classified as tubular, villous, or tubulovillous adenomas. Tubular adenomas are most common and generally have lower malignant potential, whereas villous adenomas carry greater risk and tubulovillous adenomas show intermediate risk (5). According to the US Multi-Society Task Force, adenomas ≥10 mm or with high-grade dysplasia or villous/tubulovillous histology are classified as advanced adenomas and confer substantially higher CRC risk (2).

In contrast, serrated lesions were previously regarded mainly as benign hyperplastic polyps and were not considered to have significant malignant potential. With advances in molecular pathology and endoscopic diagnosis, serrated lesions are now recognized as an important alternative pathway of colorectal carcinogenesis. According to the 5th edition of the WHO Classification (2019), serrated lesions include hyperplastic polyps (HPs), sessile serrated lesions (SSLs), and traditional serrated adenomas (TSAs) (6). HPs account for 70–80% of cases and have minimal malignant potential. SSLs account for 10–20%, are often located in the proximal colorectum, and have a high malignant risk. TSAs are rare (1%) but also carry high malignant potential (4, 7).

Notably, these two lesion types follow different carcinogenic pathways. The conventional adenoma–carcinoma pathway is characterized by chromosomal instability (CIN) and alterations involving *APC* and *KRAS* (8, 9), whereas the serrated pathway is mainly defined by *BRAF* mutation and the CpG island methylator phenotype (CIMP) (10, 11). These molecular differences suggest that risk and protective factors may act differently across lesion types. Although several shared determinants have been identified, substantial heterogeneity is evident. This review therefore systematically compares their epidemiological features, mechanisms, and differential risk and protective factors to deepen understanding of these two major colorectal precursor lesions and provide a basis for future research on colorectal cancer screening and prevention.

## 2 Literature search and review approach

This narrative review was informed by structured searches of PubMed and Web of Science from database inception through June 12, 2026. Search terms combined conventional adenoma–related terms (“conventional adenoma,” “colorectal adenoma,” and “adenomatous polyp”) and serrated lesion–related terms (“serrated lesion/polyp,” “sessile serrated lesion/polyp,” “sessile serrated adenoma/polyp,” “hyperplastic polyp,” and “traditional serrated adenoma”) with terms for the lifestyle, metabolic, and nutritional factors evaluated in this review. Reference lists of relevant reviews and eligible studies were manually screened to identify additional potentially relevant studies. Additional literature searches were conducted to supplement the evidence on the epidemiological characteristics and underlying mechanisms of conventional adenomas and serrated lesions. Detailed search strategies are provided in Supplementary Table S1.

Eligible studies included human observational studies, randomized trials, and systematic reviews or meta-analyses evaluating conventional adenomas or serrated lesions in relation to the factors of interest. Case reports, conference abstracts, editorials, and studies without relevant lesion- or exposure-specific information were excluded. CRC-only studies were not used as evidence for lesion-specific epidemiological associations but were considered when providing relevant mechanistic context. Two authors independently screened titles and abstracts for relevance and subsequently assessed the full texts of potentially eligible studies. Disagreements were resolved through discussion. Given the narrative nature of this review, formal quality assessment and quantitative evidence pooling were not performed. When individual studies reported specific serrated subtypes, including HPs, SSLs/SSA/SSPs, or TSAs, the subtype-specific terminology and findings were retained rather than generalized to serrated lesions as a whole.

## 3 Epidemiology

Conventional adenomas and serrated lesions show distinct epidemiological patterns. Conventional adenomas are the most common precancerous lesions detected during CRC screening. Age is a major determinant of adenoma occurrence. The prevalence of adenomas and advanced adenomas was higher among individuals aged 50 years or older than among those younger than 50 years (25.9% vs. 14.6 and 5.2% vs. 1.6%, respectively). A screening colonoscopy study also supported this age-related trend, showing that adenoma prevalence increased stepwise from 10.9% among individuals aged 40–44 years to 23.6% among those aged 60–64 years, accompanied by a parallel increase in advanced adenoma prevalence from 3.9 to 7.5% (12). Sex differences are also evident (12, 13), with both adenomas and advanced adenomas being more prevalent in men than in women (29.7% vs. 19.3 and 6.5% vs. 3.8%) (14).

Compared with conventional adenomas, serrated lesions appear to show a weaker age gradient. A systematic review estimated that sessile serrated polyp prevalence increased by approximately 1.9% relative per year of age, compared with 2.7% for adenomas (15). SSLs are also not uncommon in younger adults. Among 9,854 individuals undergoing index colonoscopy, adenoma prevalence was substantially lower before age 50, whereas SSL prevalence was similar in younger and older groups (7.2% vs. 6.5%) (16). In addition to age-related differences, sex distribution also appears to differ between conventional adenomas and serrated lesions. Some studies report higher serrated lesion prevalence or detection in men (17), whereas clinicopathological studies associate SSA/Ps with female sex, particularly during progression (18). In one series, the proportion of women increased from 47% in SSA/Ps without dysplasia to 59% with cytological dysplasia and 86% with invasive carcinoma (19). Thus, unlike the consistent male predominance observed for conventional adenomas, serrated lesions do not appear to exhibit a consistent sex predominance, with reported associations varying across studies.

Beyond demographic factors, geographic variation also differs between conventional adenomas and serrated lesions. For conventional adenomas, regional differences in overall prevalence appear relatively modest, with reported rates of 25.9% in Europe, 24.2% in North America, and 23.0% in Asia (14). In contrast, sessile serrated polyps/lesions show greater geographic heterogeneity, with reported prevalence rates of approximately 2.6% in Asia, 3.9% in Europe, 5.1% in the United States, and 10.5% in Australia (15). These regional differences may partly reflect variation in screening populations, colonoscopy quality, endoscopist awareness, and pathological diagnostic criteria. SSA/Ps also show a characteristic proximal colonic distribution, and their flat or sessile morphology with indistinct borders may further contribute to underdetection during colonoscopy.

## 4 Differences in carcinogenic pathways

Conventional adenomas and serrated lesions progress through distinct carcinogenic pathways (8). Conventional adenomas mainly follow the classical adenoma–carcinoma sequence, in which *APC* inactivation and subsequent activation of the Wnt/β-catenin signaling pathway are considered early and central molecular events (9). During lesion progression, additional alterations, including *KRAS* activation and inactivation of *TP53* and/or *SMAD4*, may accumulate and drive the stepwise transition from adenoma to advanced adenoma and eventually invasive CRC (20). This pathway is commonly associated with CIN and microsatellite-stable (MSS) CRC (5, 8).

In contrast, serrated lesions develop through the serrated neoplasia pathway, which is characterized by a different spectrum of molecular alterations. SSLs are frequently associated with *BRAF* mutation, activation of the MAPK signaling pathway, and CIMP (21). During progression, *MLH1* promoter hypermethylation may lead to mismatch repair deficiency and microsatellite instability-high CRC (10, 22). TSAs may involve either *KRAS* or *BRAF* mutations and represent another subtype within the serrated pathway (23). Therefore, compared with conventional adenomas, serrated lesions are more closely related to epigenetic dysregulation, DNA methylation, and mismatch repair deficiency (8, 24).

Recent single-cell transcriptomic and spatial analyses further indicate that the differences between conventional adenomas and serrated lesions extend beyond classical genetic mutations and epigenetic dysregulation. Conventional adenomas appear to be mainly driven by Wnt-mediated expansion of stem-like epithelial cells, whereas serrated lesions may arise through metaplastic programs in differentiated epithelial cells. Histological and spatial evidence further supports a bottom-up model for conventional adenomas, in which neoplastic cells expand from the crypt-base stem cell compartment. In contrast, serrated lesions appear to follow a top-down pattern, with metaplastic epithelial cells emerging near the luminal surface and progressively extending toward the crypt base (25).

The immune microenvironment may also differ between the two pathways. Some studies suggest that conventional adenomas may show reduced antigen-presentation activity and features of an immunosuppressive microenvironment. In contrast, serrated lesions may exhibit early accumulation of cytotoxic immune cells in metaplastic regions, even before the development of a high mutational burden. These immune differences suggest that tumor cell differentiation states and local immune responses may jointly shape the progression of colorectal precursor lesions (25, 26).

Overall, conventional adenomas are primarily characterized by *APC* inactivation, activation of the Wnt/β-catenin signaling pathway, stepwise genetic accumulation, and CIN. Serrated lesions, by contrast, are more strongly associated with *BRAF* or *KRAS* mutations, CpG island methylation, *MLH1* silencing, microsatellite instability, and metaplastic epithelial programs (Table 1). These mechanistic differences may partly underlie the distinct epidemiological patterns and risk factor profiles of conventional adenomas and serrated lesions.

**Table 1:** Comparison of major mechanistic features of the conventional adenoma–carcinoma and serrated neoplasia pathways.

Mechanistic dimension	Conventional adenomas	Serrated lesions
Core pathway	Normal mucosa → conventional adenoma → advanced adenoma → CRC	SSL pathway: SSL → SSL with dysplasia → CRC TSA pathway: TSA → TSA with dysplasia → CRC
Common early molecular alteration	Early *APC* inactivation with abnormal β-catenin accumulation and Wnt pathway activation	*BRAF* V600E mutations are frequent in SSLs. TSAs are molecularly heterogeneous and may harbor *BRAF* or *KRAS* mutations
Proposed epithelial program and growth pattern	Wnt-associated expansion of crypt-base stem/progenitor-like epithelial programs, consistent with a predominantly bottom-up growth model	Differentiated or metaplastic epithelial programs, with emerging evidence supporting a top-down growth model
Dominant signaling alterations	Wnt/β-catenin activation is central; *KRAS*/MAPK, TGF-β/SMAD, and *TP53*-related alterations may accumulate during progression	MAPK/ERK activation driven by *BRAF* or *KRAS* mutations is the predominant signaling feature of the serrated pathway.
Genomic and epigenetic features	Predominantly characterized by chromosomal instability and progression to MSS CRC; DNA methylation may occur during progression but is not generally considered a defining early feature.	Characterized by CIMP; *MLH1* promoter hypermethylation can lead to MMR deficiency and MSI-H CRC in a subset of SSLs, whereas TSA-associated CRCs are more often MSS.
Immune microenvironment	Emerging studies suggest relatively limited cytotoxic immune-cell infiltration and enrichment of myeloid- or regulatory T-cell-related programs in some conventional adenomas.	Emerging single-cell studies suggest relatively early enrichment of cytotoxic immune-cell populations and enhanced antigen-presentation activity in some serrated lesions.

CIMP, CpG island methylator phenotype; CRC, colorectal cancer; HP, hyperplastic polyp; MAPK/ERK, mitogen-activated protein kinase/extracellular signal-regulated kinase; MMR, mismatch repair; MSI-H, microsatellite instability-high; MSS, microsatellite stable; SSL, sessile serrated lesion; TGF-β, transforming growth factor beta; TSA, traditional serrated adenoma.

## 5 Risk and protective factors

Although conventional adenomas and serrated lesions share several risk and protective factors, current evidence suggests that their associations may differ in strength, consistency, and biological significance between the conventional adenoma–carcinoma sequence and the serrated neoplasia pathway. To better reflect these differences, the following section organizes the available evidence into three categories: factors showing clearer relevance to conventional adenomas, factors showing stronger relevance to serrated lesions, and factors associated with both lesion types or with inconclusive evidence, as summarized in Figure 1.

**Figure 1:**
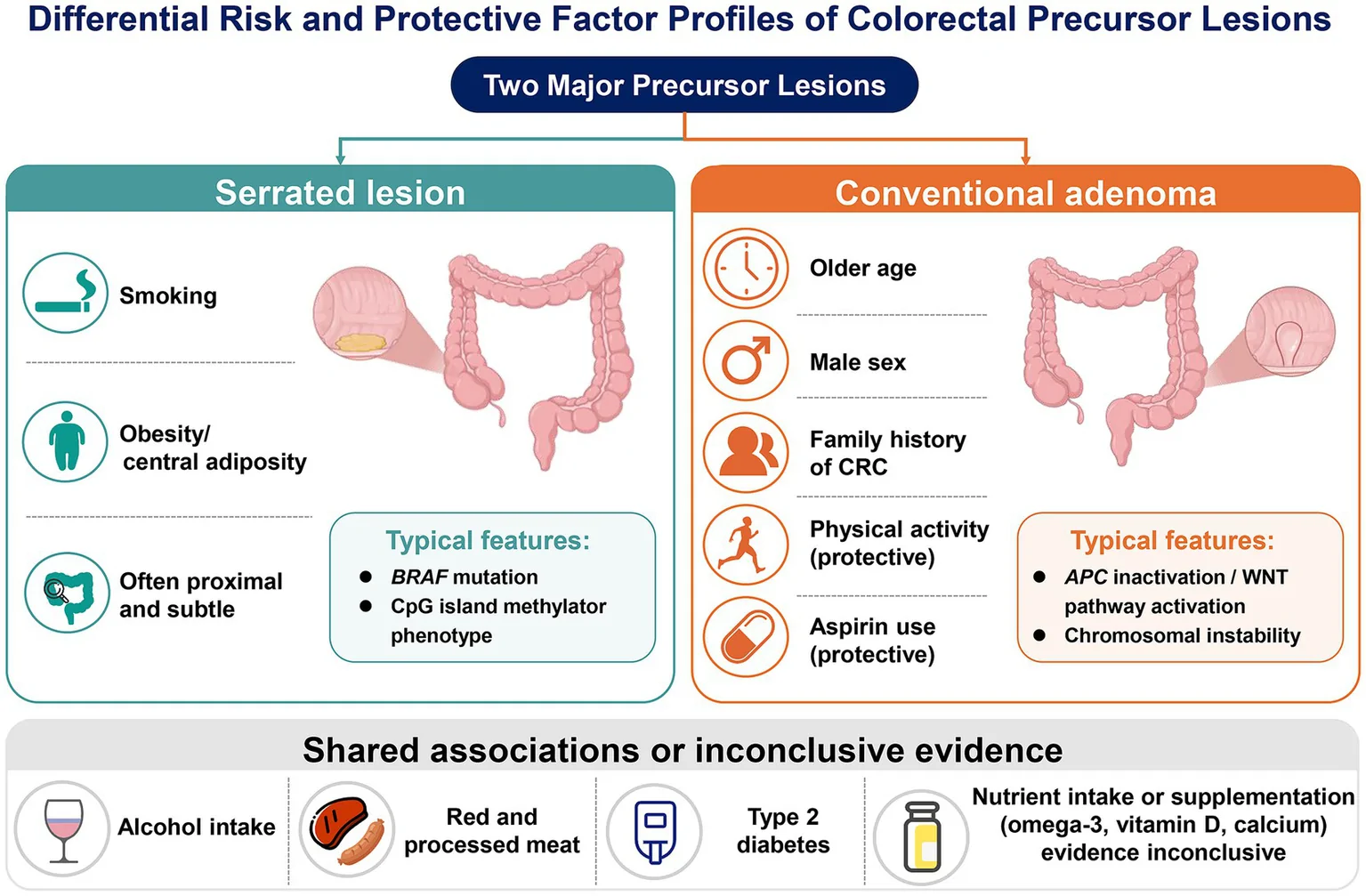
Differential risk and protective factor profiles of colorectal precursor lesions. Created in BioRender. Cai. J. (2026) https://BioRender.com/4aad2c5

### 5.1 Factors showing clearer relevance to conventional adenomas

#### 5.1.1 Family history

In addition to age and sex, family history appears to be one of the most consistently established non-modifiable risk factors for conventional adenomas. Individuals with a family history of CRC or advanced adenomas have increased risks of adenomas, advanced adenomas, and metachronous adenomas (27, 28). Ng et al. reported that siblings of patients with advanced adenomas had higher odds of adenomas than siblings of individuals with normal colonoscopy findings (OR = 3.22, 95% CI: 2.16–5.03), and approximately sixfold higher odds of advanced adenomas (29). A first-degree family history of CRC has also been associated with metachronous adenomas (OR = 1.14, 95% CI: 1.01–1.29), with stronger associations for multiple affected relatives or younger age at diagnosis (30). Associations with serrated lesions have also been reported, although findings are less consistent. In one study directly evaluating both lesion types, family history of polyps was associated with conventional adenoma recurrence (OR = 1.83, 95% CI: 1.00–3.36), but not with serrated polyp recurrence (OR = 1.41, 95% CI: 0.74–2.70) (31). Another study identified family history as an important factor associated with progression from adenoma to advanced adenoma, whereas no corresponding association was observed for progression from hyperplastic polyp to sessile serrated lesion (32). However, family history of CRC has also been associated with serrated polyps or lesions in other studies (33, 34). Overall, evidence regarding the association between family history and serrated lesions remains heterogeneous, potentially due to differences in classification, lesion definitions, and detection.

#### 5.1.2 Physical activity

Higher physical activity has been associated with a reduced risk of conventional adenomas, particularly high-risk adenomas (35). In one colonoscopy-based study, greater activity was associated with lower odds of adenomas (OR = 0.62, 95% CI: 0.43–0.89), with a stronger inverse association for high-risk adenomas (OR = 0.39, 95% CI: 0.21–0.73) (36). Activity during adolescence may also have long-term protective effects (37). In contrast, a large prospective study found that higher physical activity was inversely associated with conventional adenomas (OR = 0.87, 95% CI: 0.78–0.97), but not with serrated polyps (OR = 0.92, 95% CI: 0.82–1.04) (33). A meta-analysis similarly found no significant association between physical activity and serrated polyp risk (38). The protective association between physical activity and conventional adenomas may be partly explained by the effects of physical activity on the metabolic and inflammatory milieu that supports adenoma growth. These effects may include improved insulin sensitivity, reduced hyperinsulinemia and insulin-like growth factor signaling, decreased adiposity-related inflammation, and modulation of colonic crypt cell proliferation and apoptosis (39–41).

#### 5.1.3 Aspirin

Aspirin use also has a more established chemopreventive association with conventional adenomas. Murff et al. (42) reported that both low-dose and standard-dose aspirin use were associated with reduced risks of adenomatous polyps (OR = 0.79, 95% CI: 0.66–0.93; OR = 0.73, 95% CI: 0.58–0.90, respectively). Other studies have also shown that aspirin use is associated with reduced risks of adenoma recurrence and advanced adenoma recurrence (43–45). Mechanistically, aspirin may inhibit cyclooxygenase activity and reduce prostaglandin production, which may attenuate inflammation-driven epithelial proliferation and interfere with Wnt/β-catenin signaling involved in conventional adenoma development (46, 47). Evidence also suggests a potential protective effect of aspirin against serrated lesions. A meta-analysis reported an inverse association between aspirin use and serrated polyp risk (RR = 0.81, 95% CI: 0.67–0.99) (38). However, in a large screening study, long-term low-dose aspirin use was associated with lower adenoma detection (OR = 0.89, 95% CI: 0.80–0.99), but not with advanced serrated lesions (OR = 0.97, 95% CI: 0.72–1.32) (48). Therefore, aspirin appears to have stronger evidence supporting prevention of conventional adenomas than serrated lesions, although its use should still be evaluated according to individual risk–benefit profiles because of potential gastrointestinal adverse effects (49, 50).

### 5.2 Factors showing stronger relevance to serrated lesions

#### 5.2.1 Smoking

Smoking is a well-established risk factor for both conventional adenomas and serrated lesions, with dose–response relationships observed for smoking duration, intensity, and cumulative exposure (51–53). However, the magnitude of this association appears to be greater for serrated lesions than for conventional adenomas. Multiple studies have reported stronger associations between smoking and serrated lesions, including hyperplastic polyps and sessile serrated polyps, than between smoking and conventional adenomas (54). In pooled analyses from the Nurses’ Health Study, Nurses’ Health Study II, and Health Professionals Follow-up Study, smoking was more strongly associated with serrated polyps (OR = 2.52, 95% CI: 2.29–2.78) than with conventional adenomas (OR = 1.13, 95% CI: 1.00–1.27; *P* for heterogeneity < 0.001) (33). Similarly, Figueiredo et al. (55) reported a modest association between smoking and conventional adenomas (RR = 1.31, 95% CI: 1.08–1.58), but a substantially stronger association with serrated polyps (RR = 2.33, 95% CI: 1.76–3.07). Smoking cessation has also been associated with a reduced risk of sessile serrated lesions, further supporting a potential relationship (56).

The stronger association between smoking and serrated lesions may be related to the molecular features of the serrated pathway. Serrated lesions are commonly characterized by *BRAF* mutations, CIMP, and DNA methylation abnormalities, while smoking has been associated with CIMP-high and *BRAF*-mutated colorectal tumors (21, 22, 57). Tobacco-related carcinogens may also promote oxidative stress, DNA damage, chronic inflammation, and epigenetic alterations in the colorectal mucosa, creating a biological environment that favors the initiation or progression of serrated pathway lesions (58–60).

#### 5.2.2 Obesity

Obesity also appears to be closely associated with serrated lesions. Several adiposity-related measures have been associated with serrated polyps, with stronger associations suggested than for conventional adenomas (32, 61, 62). He et al. (33) found that, compared with individuals with a BMI < 25 kg/m^2^, those with a BMI ≥ 35 kg/m^2^ had a stronger association with serrated polyps (OR = 1.34, 95% CI: 1.23–1.46) than with conventional adenomas (OR = 1.07, 95% CI: 0.98–1.17; *P* for heterogeneity < 0.001). Bai et al. (63) similarly found that body mass index, waist circumference, and waist-to-hip ratio were associated with both lesion types, with stronger associations observed for serrated polyps. In addition, adult weight gain has been associated with increased risks of both conventional adenomas and serrated polyps, again with a stronger association for serrated polyps (64). This observed pattern may be partly attributable to obesity-related metabolic dysfunction and DNA methylation changes. Insulin resistance, adipokine dysregulation, and chronic low-grade inflammation may promote colorectal epithelial proliferation and survival (65, 66), while obesity-associated DNA methylation changes may be particularly relevant to the CIMP-driven serrated pathway (67, 68). Nevertheless, direct mechanistic evidence in serrated precursor lesions remains limited.

### 5.3 Factors associated with both lesion types or with inconclusive evidence

#### 5.3.1 Alcohol intake

Alcohol consumption has been associated with increased risks of both conventional adenomas and serrated lesions. A meta-analysis reported a positive dose–response association between alcohol consumption and conventional adenoma risk (69), including advanced adenomas in some studies (70, 71). Alcohol intake has also been linked to serrated polyps, with stronger associations reported for sessile serrated adenomas/polyps than for hyperplastic polyps (38), and possibly than for conventional adenomas (33). However, findings remain inconsistent, as some studies have observed associations only with conventional adenomas but not with serrated polyps (62). Mechanistically, alcohol may contribute to colorectal neoplasia through acetaldehyde production, oxidative stress, impaired folate metabolism, DNA damage, and inflammation (72–74). However, these mechanisms are not specific to either the conventional adenoma–carcinoma sequence or the serrated pathway. Overall, current evidence does not support a consistent preferential association with either pathway.

#### 5.3.2 Red and processed meat

High intake of red and processed meat has been associated with increased risks of both conventional adenomas and serrated lesions (38, 75–77). A meta-analysis reported that each 100 g/day increase in red meat intake and each 50 g/day increase in processed meat intake were associated with 36 and 28% higher risks of conventional adenomas, respectively (77). Red and processed meat intake has also been linked to sessile serrated lesions (75). Moreover, frying, grilling, and other high-temperature cooking methods may further increase the risk of colorectal precursor lesions by generating heterocyclic amines and polycyclic aromatic hydrocarbons, which have been associated with conventional adenomas, hyperplastic polyps, and sessile serrated lesions (78–81). Mechanistically, processed meat-derived N-nitroso compounds and mutagens generated during high-temperature cooking may contribute to colorectal neoplasia through DNA damage, oxidative stress, inflammation, and altered epithelial homeostasis (76). However, comparative evidence remains inconsistent, with some studies suggesting stronger associations with sessile serrated lesions and others finding no clear heterogeneity by lesion subtype (54, 61).

#### 5.3.3 Diabetes mellitus

Diabetes mellitus, particularly type 2 diabetes mellitus, has been associated with increased risks of colorectal precursor lesions (82). For conventional adenomas, individuals with diabetes have been reported to have a higher risk of advanced adenomas, while elevated fasting insulin levels have also been associated with increased adenoma risk, suggesting a potential role of insulin resistance and hyperinsulinemia (83, 84). Hyperglycemia may further interact with other risk factors, such as *Helicobacter pylori* infection, to increase adenoma risk (85). Evidence also supports an association with serrated lesions, as diabetes has been identified as a risk factor for sessile serrated lesions in a case–control study (86). In a large colorectal cancer screening study, type 2 diabetes was associated with increased risks of both advanced adenomas and advanced serrated polyps, with no significant difference in effect size between the two lesion types (61). These findings suggest that diabetes may contribute to colorectal precursor lesion development through broad metabolic and inflammatory mechanisms, including insulin resistance, hyperinsulinemia, chronic hyperglycemia, and systemic inflammation, while metabolic management and glycemic control may help reduce lesion risk (87).

#### 5.3.4 Nutrient intake and supplementation

Nutrient intake and supplementation show heterogeneous and generally inconclusive associations with colorectal precursor lesions. Omega-3 polyunsaturated fatty acids have been proposed to reduce colorectal neoplasia risk through anti-inflammatory effects and modulation of cyclooxygenase activity (88–90). Observational data suggest modest inverse associations between omega-3 polyunsaturated fatty acids intake and both conventional adenomas and serrated polyps, with no clear heterogeneity by lesion type (33). However, randomized trial evidence has not confirmed a significant protective effect of marine omega-3 supplementation on either conventional adenomas or serrated polyps (91).

Evidence for vitamin D and calcium is similarly inconsistent. Although some observational studies have suggested inverse associations with conventional adenomas (92, 93), randomized trials have generally failed to show a clear reduction in the risk of conventional adenomas or serrated lesions (94). Moreover, combined calcium and vitamin D supplementation has been associated with an increased risk of sessile serrated adenomas/polyps during extended follow-up in one trial (95). These discrepancies may reflect differences in study design, baseline nutrient status, supplement dose, intervention duration, and lesion classification. Overall, current evidence does not support a stable pathway-specific protective effect of omega-3 polyunsaturated fatty acids, vitamin D, or calcium on either conventional adenomas or serrated lesions, and further studies are needed to clarify their potential pathway-specific roles.

## 6 Implications for future research

The evidence summarized above suggests that conventional adenomas and serrated lesions may differ in certain epidemiological characteristics and risk-factor associations. However, much of the available evidence is observational, and the magnitude and consistency of these differences vary across studies. Despite these limitations, the distinct molecular mechanisms of the two precursor pathways, together with observed differences in risk-factor associations, point to several priorities for future research.

### 6.1 Lesion-specific considerations for future etiologic research

Conventional adenomas and serrated lesions should not necessarily be treated as a single homogeneous outcome in epidemiological studies, given their distinct molecular characteristics and potentially different risk-factor associations. Future studies should directly compare the associations of established and emerging risk factors with these two precursor lesion types and formally assess heterogeneity between them. This approach may help distinguish risk factors that are common to both lesion types from those that are more strongly associated with conventional adenomas or serrated lesions. It may also provide a more precise understanding of the potential roles of lifestyle, metabolic, and nutritional exposures across the two precursor pathways. Importantly, epidemiological associations should be investigated alongside the molecular alterations characteristic of each lesion type. Integrating exposure data with genetic, epigenetic, metabolomic, and other molecular features may help clarify the biological mechanisms underlying observed lesion-specific associations. Future studies could therefore move beyond evaluating colorectal neoplasia as a single outcome and examine whether risk-factor associations differ according to both precursor lesion type and molecular characteristics.

### 6.2 Exploring the potential of lesion-specific risk prediction

Current screening strategies and risk prediction models generally rely on established factors such as age and family history and often do not distinguish between precursor lesion types. The heterogeneity summarized in this review raises the possibility that conventional adenomas and serrated lesions may have different predictor profiles. For example, age, sex, and family history appear to be more consistently associated with conventional adenomas, whereas smoking and obesity have shown comparatively stronger associations with some serrated polyp outcomes. These differences provide a rationale for evaluating whether lesion-specific models can improve risk prediction. The distinct biological characteristics of the two precursor pathways also support investigation of molecular markers as potential complementary predictors. Future prospective studies should develop and externally validate such models and determine whether distinguishing precursor lesion types provides meaningful additional information for risk prediction.

### 6.3 Improving serrated lesion detection and classification

Accurate detection and classification of serrated lesions are essential for evaluating pathway-specific epidemiological associations. Sessile serrated lesions are particularly prone to underdetection because of their flat or sessile morphology, subtle borders, and frequent proximal location, while variability in pathological interpretation may further contribute to misclassification. Future studies should therefore examine how differences in colonoscopy quality, endoscopist performance, and pathological classification influence estimates of serrated lesion prevalence and risk-factor associations. Standardized diagnostic criteria and quality metrics, including the potential use of serrated lesion detection rate, should also be further evaluated and validated across screening settings (96, 97).

### 6.4 Development and validation of non-invasive molecular screening tools

Because fecal immunochemical test relies mainly on fecal hemoglobin detection, its sensitivity for serrated lesions may be limited, as these lesions often bleed less than conventional adenomas and are frequently located in the proximal colon (98). Therefore, non-invasive screening strategies may need to incorporate molecular markers beyond hemoglobin alone. Stool-based tests combining hemoglobin with DNA methylation, mutation, or other serrated pathway–related markers may improve the detection of sessile serrated lesions compared with fecal immunochemical test (99–102). Emerging approaches, including stool RNA markers, blood-based cell-free DNA methylation assays, microRNAs, metabolites, and exosomes, may further expand screening options (103–106). However, these methods remain insufficiently validated for serrated lesion detection. Future studies should develop and externally validate molecular panels specifically optimized for serrated pathway lesions and assess their added value in population-based screening.

### 6.5 Exploring the potential role of host-related factors in subsequent neoplasia risk

Current post-polypectomy surveillance strategies are primarily guided by established lesion characteristics, including lesion type, number, and size. Whether lifestyle and metabolic factors provide independent information on subsequent neoplasia risk beyond these established characteristics remains unclear. Future prospective studies could examine whether factors such as smoking, obesity, and metabolic status are associated with metachronous advanced neoplasia and whether these associations differ between individuals with conventional adenomas and those with specific serrated lesion subtypes. Such studies may help clarify the potential prognostic relevance of host-related factors after polypectomy and provide a basis for further investigation of their possible role in future surveillance and prevention research.

## 7 Discussion

In this review, we summarize current evidence on the epidemiological features, molecular mechanisms, and pathway-specific risk and protective factors of conventional adenomas and serrated lesions. Although the two lesion types share several epidemiological and lifestyle-related determinants, their associations with these factors differ in magnitude, consistency, and biological interpretation. Age, male sex, and family history are more consistently associated with conventional adenomas, while physical activity and aspirin use show more established protective associations with this pathway. By comparison, smoking and obesity appear to have stronger relevance to serrated lesions. Other factors, including alcohol intake, red and processed meat consumption, diabetes, and nutrient intake or supplementation, may affect both lesion types, but their pathway-specific effects remain uncertain.

The observed differences between conventional adenomas and serrated lesions may be partly related to their distinct underlying biological pathways. Conventional adenomas are mainly characterized by *APC*/Wnt signaling alterations and chromosomal instability, whereas serrated lesions are more closely associated with *BRAF*-related changes, CpG island methylation, and microsatellite instability. These findings support the consideration of conventional adenomas and serrated lesions as distinct precursor outcomes in colorectal cancer prevention research.

Earlier epidemiological research often focused on conventional adenomas or broadly defined colorectal polyps, while serrated subtypes were frequently combined rather than analyzed separately, partly because the terminology and diagnostic criteria for serrated lesions were still evolving. More recent prospective and colonoscopy-based studies have directly compared conventional adenomas and serrated lesions, demonstrating heterogeneity in their associations with lifestyle, metabolic, and dietary factors (32, 33, 54, 63). A recent systematic review and meta-analysis further quantified several subtype-specific associations (107). Nevertheless, previous research has rarely considered epidemiological factors together with the distinct molecular characteristics of conventional adenomas and serrated lesions. The present review therefore considers epidemiological associations of risk and protective factors alongside the molecular and biological characteristics of the two precursor lesion types. A key contribution of this review is to bring these two lines of evidence into a common comparative framework, allowing the observed differences in risk-factor associations and their potential biological basis to be considered together. This integrative perspective may contribute to a more comprehensive understanding of the similarities and differences between the two major colorectal precursor pathways.

Several limitations should be considered. This review is a narrative synthesis rather than a systematic review or meta-analysis; therefore, formal quality assessment and quantitative evidence pooling were not performed. In addition, differences in serrated lesion terminology and classification, endoscopic detection and pathological interpretation, study populations, and exposure assessment may limit comparability across studies. Much of the available evidence is observational, and direct comparisons of conventional adenomas and serrated lesions within the same study populations remain relatively limited. Accordingly, the reported differences in risk-factor associations between the two pathways should be interpreted with caution. Future prospective studies directly comparing different lesion types within the same populations and integrating epidemiological, molecular, pathological, and endoscopic data may help clarify the consistency of these associations and their potential relevance to future risk assessment and prevention research.
