# Supplementary material for: Differential risk and protective factors for conventional adenomas and serrated lesions: implications for colorectal cancer prevention
Source: Front Public Health. 2026 Oct 14;14:1949376. doi: 10.3389/fpubh.2026.1949376 (PMC13715656; doi:10.3389/fpubh.2026.1949376)
Supplement: Supplementary file 1 [file Table_1.DOCX]

**Supplementary Table S1. Search strategies used in PubMed and Web of Science**

| **Search category** | **Search strategy** |
| --- | --- |
| **1. Core search**  *Risk and protective factors* | ( "conventional adenoma" OR "colorectal adenoma" OR "adenomatous polyp" ) AND ( "serrated lesion" OR "serrated polyp" OR "sessile serrated lesion" OR "sessile serrated polyp" OR "sessile serrated adenoma" OR "hyperplastic polyp" OR "traditional serrated adenoma" ) AND ( "risk factor" OR "protective factor" OR lifestyle OR metabolic OR diet ) |
| **2. Factor-specific search**  *Individual lifestyle, metabolic, and nutritional factors* | ( ( "conventional adenoma" OR "colorectal adenoma" OR "adenomatous polyp" ) OR ( "serrated lesion" OR "serrated polyp" OR "sessile serrated lesion" OR "sessile serrated polyp" OR "sessile serrated adenoma" OR "hyperplastic polyp" OR "traditional serrated adenoma" ) ) AND ( "family history" OR smoking OR obesity OR "physical activity" OR aspirin OR alcohol OR "red meat" OR "processed meat" OR diabetes OR "omega-3" OR "vitamin D" OR calcium ) |
| **3. Supplementary search**  *Epidemiological characteristics and underlying mechanisms* | ( "conventional adenoma" OR "colorectal adenoma" OR "adenomatous polyp" ) AND ( "serrated lesion" OR "serrated polyp" OR "sessile serrated lesion" OR "sessile serrated polyp" OR "sessile serrated adenoma" OR "hyperplastic polyp" OR "traditional serrated adenoma" ) AND ( epidemiolog OR prevalence OR incidence OR distribution OR mechanism OR molecular OR pathway ) |

The same Boolean keyword combinations were applied in PubMed and Web of Science. Both databases were searched from inception through June 12, 2026.
